# *Malassezia restricta* Pneumonia in Solid Organ Transplant Recipients: First Report of Two Cases

**DOI:** 10.3390/jof7121057

**Published:** 2021-12-10

**Authors:** Alessandra Mularoni, Elena Graziano, Alice Annalisa Medaglia, Barbara Buscemi, Taylor Eddens, Lavinia Martino, Daniele Di Carlo, Antonio Cascio, Pier Giulio Conaldi, Alessandro Bertani, Paolo Antonio Grossi

**Affiliations:** 1 Department of Infectious Diseases, IRCCS-ISMETT (Mediterranean Institute for Transplantation and Advanced Specialized Therapies), 90127 Palermo, Italy; amularoni@ismett.edu; 2 Division of Infectious Diseases, Department of Medicine, University of Udine, Azienda Sanitaria Universitaria Friuli Centrale (ASUFC), 33100 Udine, Italy; 3 Infectious and Tropical Disease Unit, AOU Policlinico “P. Giaccone”, 90127 Palermo, Italy; alicemedaglia@gmail.com; 4 Department of Nephrology, IRCCS-ISMETT (Mediterranean Institute for Transplantation and Advanced Specialized Therapies), 90127 Palermo, Italy; bbuscemi@ismett.edu; 5 Division of Allergy/Immunology, UPMC Children’s Hospital of Pittsburgh, Pittsburgh, PA 15224, USA; eddenstj@gmail.com; 6 Department of Pulmonology, IRCCS-ISMETT (Mediterranean Institute for Transplantation and Advanced Specialized Therapies), 90127 Palermo, Italy; lmartino@ismett.edu; 7 Department of Laboratory Medicine and Advanced Biotechnologies, IRCCS-ISMETT (Mediterranean Institute for Transplantation and Advanced Specialized Therapies), 90127 Palermo, Italy; ddicarlo@ismett.edu (D.D.C.); pgconaldi@ismett.edu (P.G.C.); 8 Infectious Diseases Unit, Internal Medicine and Medical Specialties, Department of Health Promotion, Mother and Child Care, University of Palermo, 90127 Palermo, Italy; antonio.cascio03@unipa.it; 9 Department of Thoracic Surgery, IRCCS-ISMETT (Mediterranean Institute for Transplantation and Advanced Specialized Therapies), 90127 Palermo, Italy; abertani@ismett.edu; 10 Infectious and Tropical Diseases Unit, Department of Medicine and Surgery, University of Insubria, 21100 Varese, Italy; paolo.grossi@uninsubria.it

**Keywords:** lung transplant, kidney transplant, emerging fungal infections, *Malassezia restricta*

## Abstract

Emerging fungal infections are a major challenge in solid organ transplantation (SOT) and are associated with high morbidity and mortality. We report two cases of *Malassezia restricta* pneumonia in SOT recipients. Infections were diagnosed with molecular analysis and histology. Patients were treated with antifungal therapy and have fully recovered.

## 1. Introduction

The high rate of invasive fungal infections (IFI) in solid organ transplant (SOT) is the result of the profound effects of immunosuppressive agents on host–pathogen interaction mechanisms [1,2]. Besides well-characterized pathogenic fungi such as *Aspergillus* and Pneumocystis, more recently a wide range of infections with rare fungal species that can be relatively innocuous in an immunocompetent host have been described. Collectively, these emerging IFI account for 7–10% of all fungal infections in SOT-patients [2]. Though rare, these infections are often associated with a high rate of morbidity and mortality [1]. Among these rare pathogens, *Malassezia* spp., a normal host of skin flora, is an emerging cause of invasive infections in immunocompromised hosts. We describe two clinical cases of *Malassezia restricta* pneumonia in SOT. These patients had a successful outcome, highlighting the importance of considering rare IFI in SOT recipients and of applying all the appropriate diagnostic and therapeutic strategies.

## 2. Case Reports

### 2.1. Case 1

**Figure 1:**
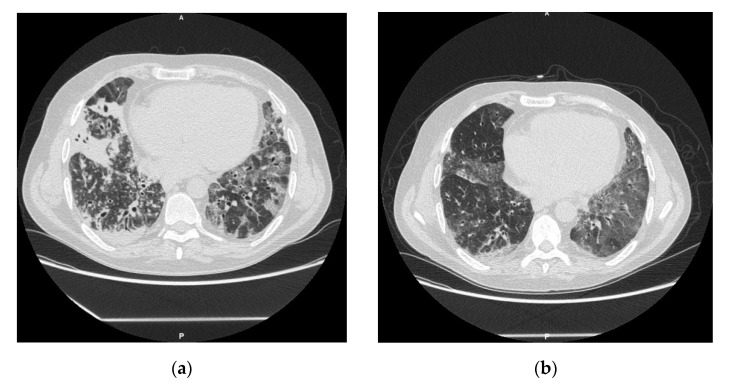
(**a**) Presentation CT scan of Case 1; (**b**) CT scan after antifungal treatment.

A 43-year-old male with idiopathic pulmonary fibrosis underwent double-lung transplant (LT) at our institution in July 2014. His immunosuppressive regimen included tacrolimus, mycophenolate mofetil, and prednisone. His early post-LT course was complicated by one episode of humoral rejection (at 17 months post-LT) treated with high dose steroid therapy, one episode of acute cellular rejection (at 19 months post-LT) treated with thymoglobulin at the dosage of 125 mg per day for 6 days, and a persistent acute cellular rejection a month later treated with another course of thymoglobulin at the same dosage. During these treatments, he also received pre-emptive valganciclovir in consideration of minimal Cytomegalovirus (CMV) blood replication. Then, 22 months after-LT, he required admission for fever and bilateral pneumonia. A chest computed tomography (CT) showed persistent ground glass opacities, with tree-in-bud appearance, of the right middle and lower lobes (Figure 1a). Laboratory results showed an elevated C-reactive protein of 78.8 mg/L (normal range 0–5 mg/L) and a normal white blood cells count. Blood cultures turned negative. The forced expiratory volume in one second (FEV1) was 1.73 L and the forced vital capacity (FEV1) of 2.16 L. Empirical antibiotic and antifungal therapy with meropenem, linezolid, and liposomal amphotericin B was started with mild clinical improvement. A broncho alveolar lavage (BAL) showed no growth on bacterial and fungal cultures, and galactomannan on BAL was negative. Lung transbronchial biopsy (TBB) showed no signs of acute rejection but was notable for acute bronchitis with aggregates of round and oval elements suggestive for fungi at the PAS staining. PCR 18S-sequencing was performed on the fresh tissue and identified *Malassezia restricta*. The patient was then started on voriconazole, with a significant progressive improvement in respiratory function, with an increase in both FEV 1 (2 L) and FVC (2.55 L). During the antifungal treatment, the tacrolimus dosage was adjusted according to the serum concentration and aimed at trough levels of 8–10 ng/mL. After 12 months, a new lung-TBB (36 months after-LT) showed the persistence of *Malassezia restricta* on PCR 18S-RNA molecular identification. At an 18-month follow up (42 months after-LT), both lipid-enriched culture and 18S-RNA on biopsy were negative for fungal pathogens, and the chest-CT-scan showed a notable improvement of radiographic findings (Figure 1b). At a 2-year follow up (4 years after-LT), voriconazole was discontinued and the patient maintained a stable respiratory function. He had no infection recurrence at his 3-year follow up.

### 2.2. Case 2

**Figure 2:**
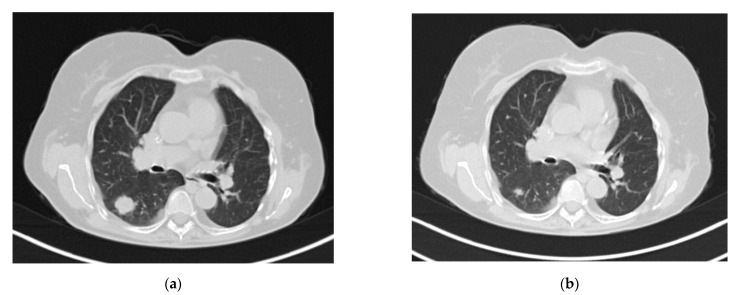
(**a**) Presentation CT scan of Case 2; (**b**) CT scan after antifungal treatment.

A 52-year-old female presented with a lung nodule in the right lower lobe one year after living related donor kidney transplant (KT) for nail-patella syndrome in February 2020. Her post-KT course was uncomplicated, and her immunosuppressive regimen was based on tacrolimus, mycophenolate mofetil, and steroid. On a routine CT scan, a solitary pulmonary nodule with a small satellite lesion was accidentally found at 2 months post-KT (Figure 2a). A positron emission tomography (PET) revealed high 18-Fluorodeoxyglucose-avidity with a value of standardized uptake value (SUV) of 12.3. The patient did not show any respiratory symptoms and the physical examination and laboratory results were unremarkable. Blood cultures were not performed as the patient was afebrile and the main diagnostic hypothesis was the neoplastic disease. To rule out a malignant aetiology, a CT guided transthoracic needle biopsy was performed. Histologic analysis of the specimen found granulomatous inflammation with multinucleated giant cells containing oval forms positive on PAS and Grocott staining. After deparaffination of the tissue section, we performed PCR 18S-RNA-sequencing. 18S-sequencing analysis was most consistent with the genus and species *Malassezia restricta*. After three-month antifungal therapy with itraconazole, a follow up CT scan showed a complete resolution of the lung nodule (Figure 2b). Due to pharmacokinetic interaction between itraconazole and calcineurin inhibitors, frequent adjustments of tacrolimus dosage were needed based on through levels (max 19 ng/mL–min 7.1 ng/mL) to avoid nefrotoxicity. Renal function remained constant without any rise of creatinine levels. Maintenance therapy with oral itraconazole was continued for a month further.

## 3. Comments

These two cases report the unique presentation of pulmonary *Malassezia restricta* in SOT recipients while offering insight into the diagnostic and therapeutic challenges with regard to rare IFI. In immunocompetent patients, *Malassezia* classically presents with cutaneous infections [3] and, more recently, with more invasive forms of infections such as endocarditis [4]. In the presence of certain risk factors, including the use of broad-spectrum antibiotic, total parenteral nutrition, neonates and preterm infants, and prolonged immunosuppression, disseminated *Malassezia* infection can occur in patients with colonized skin [5]. *Malassezia* is prone to forming biofilms and, accordingly, many cases of disseminated *Malassezia* infections occurred in the presence of a central venous catheter (CVC) [6,7]. Our patients were both immunosuppressed as a result of the anti-rejection therapy and, interestingly, were both dog owners. Dogs can be the source of the infection, as described by Chang et al. [8]. Blaes et al. described four cases of pulmonary infections caused by *Malassezia furfur* in stem cell transplant recipients, with complete resolution in three cases and one persistent infection resulting in pulmonary hemorrhage and death. Notably, the latter case was severely neutropenic (0.1 cells/microL at the time of infection) [9]. The diagnosis of *Malassezia* is challenging as it does not grow in typical fungal media; it requires specific lipid supplementation (such as sterile olive oil), prolonged incubation of blood cultures and serum 1,3-β-D-glucan is not elevated even in the presence of fulminant fungemia [10]. This might explain the culture negativity of the bronchoalveolar lavage of our first case. Here, we report the use of 18S-sequencing on tissue specimens which, coupled with the histologic analysis suggestive of IFI, allowed a swift diagnosis and therapeutic management. Delayed or inadequate treatment is associated with negative outcomes in both immunocompetent and immunocompromised patients [4,9]. The standard treatment consists of voriconazole, amphotericin B, or itraconazole [10,11]. *Malassezia* bloodstream infections can also be treated solely by removal of the infected CVC, which was not an option in this case [12]. To the best of our knowledge, these cases are the first description of primary pulmonary *Malassezia* infections in SOT who did not have an indwelling CVC or TPN. In summary, the presented clinical cases illustrate many critical aspects of the diagnosis and management of SOT-recipients with rare IFI. First, these cases demonstrate the importance of keeping emerging fungal species in the differential diagnosis in immunosuppressed SOT-recipients and highlight the need for further investigation of these rare pathogens. Second, these cases underscore the utility of molecular techniques to reach a diagnosis that may otherwise be challenging. Finally, these cases report the unique clinical manifestation, diagnostic tools, and management options of rare fungal pathogens as a reference for future cases.

## 4. Conclusions

We report the first two documented cases of *Malassezia restricta* pneumonia in a LT-recipient and a KT-recipient without the typical risk factors for invasive *Malassezia* infection, diagnosed with the support of molecular diagnostic techniques.

## Data Availability

Anonymized clinical data are available at request.
